# Sevoflurane and desflurane effects on early cognitive function after low‐risk surgery: A randomized clinical trial

**DOI:** 10.1002/brb3.3017

**Published:** 2023-04-21

**Authors:** Vilma Kuzminskaite, Egle Kontrimaviciute, Evaldas Kauzonas, Ieva Slauzgalvyte, Greta Bukelyte, Greta Bruzyte‐Narkiene, Dalius Jatuzis

**Affiliations:** ^1^ Faculty of Medicine Clinic of Anesthesiology and Intensive Care Institute of Clinical Medicine Vilnius University Vilnius Lithuania; ^2^ Faculty of Medicine Vilnius University Vilnius Lithuania; ^3^ Faculty of Medicine Clinic of Neurology and Neurosurgery Institute of Clinical Medicine Vilnius University Vilnius Lithuania

**Keywords:** anesthesia, desflurane, postoperative cognitive dysfunction, sevoflurane

## Abstract

**Background and objectives:**

Deleterious effects on short‐term and long‐term quality of life have been associated with the development of postoperative cognitive dysfunction (POCD) after general anesthesia. Yet, the progress in the field is still required. Most of the studies investigate POCD after major surgery, so scarce evidence exists about the incidence and effect different anesthetics have on POCD development after minor procedures. In this study, we compared early postoperative cognitive function of the sevoflurane and desflurane patients who experienced a low‐risk surgery of thyroid gland.

**Materials and methods:**

Eighty‐two patients, 40 years and over, with no previous severe cognitive, neurological, or psychiatric disorders, appointed for thyroid surgery under general anesthesia, were included in the study. In a random manner, the patients were allocated to either sevoflurane or desflurane study arms. Cognitive tests assessing memory, attention, and logical reasoning were performed twice: the day before the surgery and 24 h after the procedure. Primary outcome, magnitude of change in cognitive testing, results from baseline. POCD was diagnosed if postoperative score decreased by at least 20%.

**Results:**

Median change from baseline cognitive results did not differ between the sevoflurane and desflurane groups (–2.63%, IQR 19.3 vs. 1.13%, IQR 11.0; *p* = .222). POCD was detected in one patient (1.22%) of the sevoflurane group. Age, duration of anesthesia, postoperative pain, or patient satisfaction did not correlate with test scores. Intraoperative temperature negatively correlated with total postoperative score (*r* = –0.35, *p* = .007).

**Conclusions:**

Both volatile agents proved to be equivalent in terms of the early cognitive functioning after low‐risk thyroid surgery. Intraoperative body temperature may influence postoperative cognitive performance.

## Graphical abstract

Sevoflurane and desflurane equally affects early cognitive functioning after minor surgery. Significant decline in postoperative performance (>20%) was mostly common in memory tasks. Intraoperative temperature may affect postoperative cognitive function.

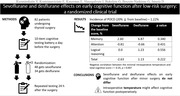

## 1 INTRODUCTION

Rather broad definition of postoperative cognitive dysfunction (POCD) is used in scientific literature. Usually, it is described as a newly occurring cognitive impairment that develops after anesthesia and lasts from several days to months (Rundshagen, 2014). A lack of unified definition and diagnostic criteria makes POCD an overly complex and puzzling diagnosis with vast differences in reported prevalence, ranging from 17% to 56% (Czyż‐Szypenbajl et al., 2019).

Recent studies identify POCD as one of the major hindrances for patients to return to a normal domestic environment due to marked impairments observed in their information acquisition, language functions, abstract thinking, situational judgment, and problem‐solving skills (Czyż‐Szypenbajl et al., 2019; Shoair et al., 2015). Despite additional evidence showing that POCD is likely to prolong hospital stay, it continues to lack appropriate recognition by anesthesia providers and surgical teams (Shoair et al., 2015; Wang et al., 2014).

The current evidence on whether the use of inhalational anesthetics is an important factor in POCD development is controversial and often low class of evidence (Huang et al., 2023). On the one hand, compared to the type of surgery or anesthesia, patient‐related factors like age or preexisting impairment in cognition are considered of major importance (Czyż‐Szypenbajl et al., 2019; Evered et al., 2011; Guay, 2011). On the other hand, volatile anesthetics, and surgery itself have been associated with the disruption of the blood–brain barrier through inflammatory pathways (Huang et al., 2023; Nakao et al., 2019; Wu et al., 2012). Inflammatory hypothesis of POCD is supported by some recent studies showing beneficial effect of steroids or probiotics in reducing cognitive impairment postoperatively (Sakic et al., 2023; Wang et al., 2021).

Even though volatile anesthetics have been associated with cognitive conditions, such as delirium (Saller et al., 2022), they are still often used for maintenance of general anesthesia due to relatively low costs. Sevoflurane and desflurane are among the “youngest” and most commonly used volatile agents. Despite belonging to the same pharmacological group, several clinical differences had been observed between the two anesthetics. Desflurane is widely recognized for its faster emergence time, better vigilance, and improved patient satisfaction (Chen et al., 2015). Moreover, it may display less adverse qualities compared to other inhalational anesthetics in the clinical setting (Rasmussen & Steinmetz, 2015; Vlisides & Xie, 2012). Unfortunately, there remains a lack of randomized clinical trials comparing the effects of different volatile agents. Mostly such POCD studies focus on major surgery or use improper instruments to diagnose subtle changes in cognition. So, surprisingly little is known about cognitive impairment after low‐risk procedures.

Thyroid surgery is an example of a minor surgical risk procedure (Kristensen et al., 2014). In our region, thyroid disease is common among the population: in 2019 almost 900 operations on thyroid gland had been performed in Lithuania (32.2 cases per 100,000) (Lithuanian Ministry of Health, Health Information Centre of Institute of Hygiene, 2020).

Bearing that in mind, to aid in closing this previously described evidence gap in POCD research, we present the findings of a randomized study that compares the effects general anesthesia maintained with either sevoflurane or desflurane has on early postoperative cognitive function after thyroid surgery.

Since desflurane has desirable effects on postoperative emergence, we hypothesize that desflurane is associated with less cognitive impairment than sevoflurane.

## 2 MATERIALS AND METHODS

### 2.1 Study design and participants

This prospective, randomized, double‐blinded study was performed at Vilnius University Hospital Santaros Klinikos (Vilnius, Lithuania) over a two‐year period (December 1, 2017 to December 1, 2019). The approval for the trial was obtained from the Vilnius Regional Biomedical Research Ethics Committee (Approval No. 158200‐17‐949‐453; Date: September 12, 2017). All eligible patients were provided with the information about the study and signed an informed consent form on the day of inclusion.

**FIGURE 1:**
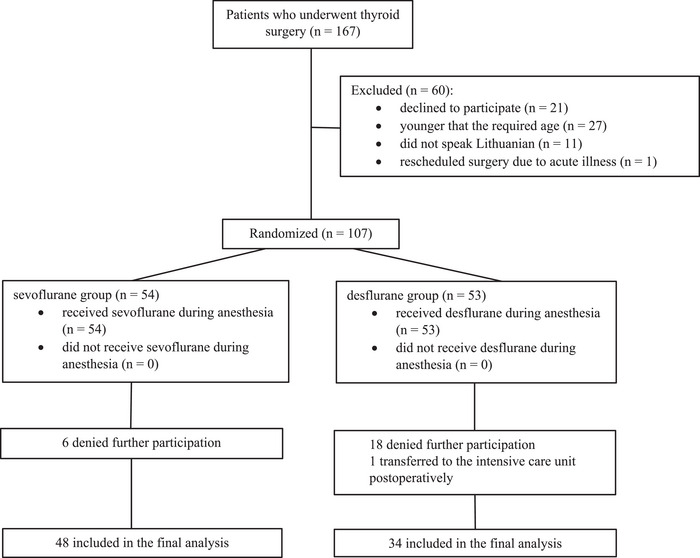
CONSORT flowchart of study participants.

Patients were invited to take part in the trial if they fit the following criteria: elective thyroid surgery under general anesthesia, hospitalization at least one day before the surgery and age ≥40 years. Those who refused to participate or continue participation, did not speak Lithuanian, or had been prediagnosed with neurological, psychiatric, or cognitive disorder were excluded from the study. Participants were also eliminated from the study if they were discharged less than 24 h after the surgery (Figure 1).

### 2.2 Allocation and randomization of the study participants

Random allocation into sevoflurane and desflurane study arms was performed using a pool of sealed opaque envelopes containing an equal number of sevoflurane and desflurane codes. In the operating room just before the induction of anesthesia the attending anesthesiologist was asked to draw one envelope and unsealed it without disclosing the code to the researcher. The anesthesiologist did not belong to the research team and did not have access to cognitive scoring results. The attending anesthesiologist was also asked to enter the code of volatile anesthetic into the intraoperative data collection sheet.

Both the patient and the researchers responsible for allocation and cognitive testing were blind to group allocation results. To avoid unblinding, assessment of baseline cognitive tests, postoperative retesting and collection of intraoperative data were assigned to separate members of the research team.

### 2.3 Anesthesia management

The anesthesia management was left to the discretion of the anesthesiologist in charge, except for the requirement to keep entropy between 40 and 60. Episodes of hypotension or desaturation were obligatory to record, as well as the lowest intraoperative body temperature measured in the nasopharynx.

### 2.4 Psychometric testing and postoperative evaluation

Baseline cognitive status was determined using cognitive testing one day before the surgery. Additionally, severe preoperative anxiety and depression was detected using the Hospital Anxiety and Depression Scale (HADS) (≥11 points were considered abnormal). The tests were performed prior to any sedative premedication in a separate quiet hospital room. Assessment was done by the same team of two trained investigators. Both baseline and postoperative cognitive evaluations for a separate participant was done by the same investigator.

The cognitive tasks involved three domains: memory, attention, and logical reasoning. A 10‐item test battery consisted of Auditory Verbal Learning (memory), Odd One Out (logical reasoning), Sequence of Numbers (logical reasoning), Digit Span (memory), Backwards Spelling (attention), Math Processing (logical reasoning), Trail‐Making Test A and B (attention), Cross‐Out Paper and Pencil (attention), and 1‐Back Tests (attention). The total maximum score was 135 points, with each of the 3 categories worth 45 points. The patients were retested 24 h postoperatively with the same modified set of tests. Primary outcome was defined as a magnitude of change in cognitive testing results (total and in separate cognitive domains) from baseline 24 h after the surgery in both study groups. Secondary outcome was incidence of POCD 24 h postoperatively. POCD was defined as more than 20% decrease from the total baseline score.

Postoperative pain score was based on 0 to 10 points Numerical Pain Scale (NPS) (0—no pain, 10—worst pain imaginable). Satisfaction after the surgery was assessed using a Likert‐type scale (1—not at all satisfied, 5—extremely satisfied).

### 2.5 Statistical analysis

Statistical analysis of the data was performed with SPSS v23.0 and XLSTAT 2018 software. Normality of the data was verified using the Shapiro–Wilk test.

Since previous studies POCD studies focus on the major rather than low‐risk surgery, it was difficult to set the appropriate expected cognitive testing target differences in the sample size calculations. We assumed for the 10% intergroup difference in postoperative score decline (15% and 25% of the two groups) and a dropout ratio of 10%, so to achieve the 5% significance level and 80% power, 55 patients should be included in each of the study groups.

Depending on the normality of data, the *T*‐test, ANOVA, Mann–Whitney *U*, or the Kruskal–Wallis test was conducted to compare the continuous preoperative and intraoperative variables of the sevoflurane and desflurane groups. Correspondingly, Fisher's exact or *χ* ^2^ test was used for intergroup analysis of categorical data and incidence of POCD. The difference between the study groups on the primary outcome was calculated with the *T*‐test or Mann–Whitney *U* test, based on the normality of data.

Comparison of baseline and postoperative cognitive score was performed using the Paired Sample *T*‐test (if the data distribution was normal) or the Wilcoxon signed‐rank test (if the data was nonnormally distributed). Respectively, the relationship between perioperative variables and cognitive scores was measured with Pearson's or Spearman's correlations. The correlation was considered strong if *R* value was more than –0.7, moderate if –0.3 < *r* < –0.7, and weak if *r* < –0.3. The results of all tests were considered statistically significant, if a two‐tailed *p* value was less than .05.

## 3 RESULTS

### 3.1 Characteristics of study participants

Out of 167 patients who were eligible to participate in our study, 82 participants were enlisted in the final analysis, with 48 and 34 patients in the sevoflurane and desflurane groups, respectively. The majority of the patients (82.92%) were female, while the median age of the participants reached 52.5 years.

Analysis of demographic aspects of the groups did not reveal any significant differences. Perioperative parameters, such as duration of anesthesia, opioid consumption, levels of postoperative pain, and overall satisfaction were also comparable (Table 1).

**TABLE 1:** Demographic and perioperative data by groups

	Sevoflurane	Desflurane	Sevoflurane	Desflurane	
Variable	*N* (%)		Median (IQR)		*p* Value*
Sex					
Male	9 (18.75%)	5 (14.71%)			.632
Female	39 (81.25%)	29 (85.29%)			
Age in years			52.5 (20.5)	52.0 (27.0)	.359
BMI, kg/m^2^			27.18 (6.8)	28.58 (9.29)	.937
Education					
Secondary	8 (16.67%)	8 (23.53%)			.634
College	14 (29.17%)	15 (44.12%)			.336
University	20 (41.67%)	11 (32.35%)			.178
N/A	6 (12.5%)	0 (0.0%)			
Smoking	14 (29.17%)	10 (29.41%)			.819
Use of benzodiazepines	8 (16.67%)	3 (8.82%)			.211
Depression	3 (6.25%)	0 (0.0%)			.130
Severe anxiety	7 (14.58%)	3 (8.82%)			.238
Duration of surgery, min			120.0 (51.25)	120.0 (37.5)	.877
Duration of anesthesia, min			147.5 (43.75)	150.0 (36.25)	.615
Fentanyl dose, μg/kg/min			1.59 (0.73)	1.83 (0.96)	.279
Morphine dose, mg/kg/h			0.03 (0.05)	0.02 (0.05)	.294
Lowest intraoperative temperature, °C			36.2 (0.6)	36.1 (0.4)	.321
Preoperative logic score, pts			37.5 (5.63)	36.75 (6.38)	.968
Preoperative attention score, pts			30.62 (7.12)	31.69 (7.49)	.500
Preoperative memory score, pts			26.68 (13.74)	29.97 (15.95)	.431
Total preoperative cognitive score, pts			94.86 (27.15)	100.85 (20.84)	.171
Postoperative satisfaction, pts			4.0 (1.0)	4.0 (1.0)	.923
Postoperative pain, pts			2.0 (3.0)	3.0 (3.0)	.797

Abbreviation: BMI—body mass index, IQR—interquartile range, min—minutes, *N*—number of participants, pts—points.

* *p* Values were calculated using *T*‐test, ANOVA, Mann–Whitney *U*, the Kruskal–Wallis tests, Fisher's exact or *χ* ^2^ test, depending on the type (numerical or categorical) and normality of the data.

### 3.2 Cognitive testing results

Preoperative total cognitive test score was slightly, yet not significantly, greater in the desflurane group (100.85, IQR 20.46 vs. 94.86, IQR 27.15; *p* = .171).

No intergroup difference was detected regarding the change from total baseline cognitive score: −2.63% (IQR 19.3) in sevoflurane group vs. 1.13% (IQR 11.0) in desflurane group, *p* = .222; nor did the groups differ when separately accounting for a change in memory, attention, or logical reasoning scores (Table 2).

**TABLE 2:** Median change from baseline cognitive scores by groups

Score change	Sevoflurane group	Desflurane group	*p* Value*
Memory, %	2.6 (IQR 38.63)	6.87 (IQR 34.54)	.340
Attention, %	–0.81 (IQR 16.18)	0.66 (IQR 19.44)	.431
Logical reasoning, %	0.0 (IQR 14.27)	1.23 (IQR 14.54)	.556
Total, %	–2.63 (IQR 19.30)	1.13 (IQR 11.00)	.222

Abbreviation: IQR—interquartile range.

* *p* Values were calculated using the independent samples *T*‐test or Mann–Whitney *U* test.

Similarly, there was no significant postoperative total performance score difference observed between the sevoflurane and desflurane groups: 97.46 (IQR 25.36) vs. 102.11 (IQR 20.84), *p* = .131.

One patient (1.22%) belonging to the sevoflurane (41‐year‐old female) group fit our diagnostic criterion for POCD. Even though the median preoperative and postoperative cognitive scores in the total study population were nearly identical (98.33, IQR 22.45 vs. 98.71, IQR 25.32; *p* = .806), decline of 20% was seen in memory (*n* = 15; 18.29%), attention (*n* = 6; 7.31%), and logical reasoning (*n* = 5; 6.09%) scores of some patients.

### 3.3 Impact of perioperative factors on cognitive scores

The total postoperative scores did not correlate with age, duration of anesthesia, intraoperative opioid administration, postoperative pain, or patient satisfaction scores. Weak positive correlations were observed between age and attention score (*r* = 0.26, *p* = .021), as well as BMI and memory scores (*r* = 0.25, *p* = .032). Even though men had lower median preoperative cognitive score than women, the difference was insignificant (87.81 vs. 96.88, *p* = .064). Furthermore, the change from the total baseline cognitive score also did not differ between both sexes: −1.41% in males and 0.21% in females (*p* = .581), nor did the results of memory, attention, or logical reasoning tasks.

Negative correlations were found between the lowest intraoperative temperature and total postoperative score, as well as memory score (total: *r* = −0.35, *p* = .007; memory: *r* = −0.3, *p* = .020). In particular, the importance of intraoperative temperature seemed to be higher in the sevoflurane group (total: *r* = −0.46, *p* = .018; memory: *r* = −0.44, *p* = .027). A slight negative correlation between anesthesia duration and postoperative logic score (*r* = −0.24, *p* = .028) was also present and was also more pronounced in the sevoflurane group (*r* = −0.3, *p* = .039). Analysis of the desflurane group did not reveal any significant correlations.

## 4 DISCUSSION

Even though not statistically significant, sevoflurane group's postoperative results decreased on average by 1.25% whereas the patients receiving desflurane demonstrated a slight increase in postoperative score (1.61%). Despite that, this study revealed no statistical difference in cognitive scores between the sevoflurane and desflurane groups. Our data corresponds with the most recent review carried out by Alalawi and Yasmeen (2018), where cognitive recovery pattern was similar in both groups. Then again, this review includes only three observational studies with a moderate sample size (60–70) of elderly (>65 years) patients undergoing a various elective procedures and cognitive testing mostly done using the Mini‐Mental State Exam, which is not sensitive enough to capture subtle changes in cognitive functioning.

Learning or practice effects are not uncommon as well when repeated tests are performed (Berger et al., 2015). To mitigate the above‐mentioned effects, we repeated the testing procedure only once by using the two modified sets of psychometric tasks. Diminished preoperative anxiety and stress could also partially contribute to the improved postoperative scores.

Due to the lack of uniform definition, testing methods, diagnostic criteria, and a wide variety of investigated time frames and surgery types, the frequency of postoperative cognitive decline is exceptionally difficult to interpret. In our study only one patient (1.22%) was diagnosed with POCD. Advanced age (> 65 years), age‐related comorbidities, and degenerative changes predispose a cognitive decline by increasing vulnerability to neurological insults (Alalawi & Yasmeen, 2018; Moller et al., 1998; Monk et al., 2008). Therefore, rather low incidence of POCD and lack of association between age and perioperative scores could be partially explained by our relatively young study population.

Interestingly, our study showed that the pronounced decline (> 20%) from the baseline was much more commonly found in memory tasks (18.29%). Even in normal aging, the inflammatory processes of the central nervous system intensify, and the impaired function of microglia cells can cause behavioral and cognitive consequences. Memory as well as attention are the basic most affected cognitive functions (Glisky, 2007; Norden & Godbout, 2013). The experimental trials reveal that volatile anesthetics are likely to activate TREK‐1 potassium channels in the brain that are involved in memory impairment. Yet, the exact mechanism is still unknown. Furthermore, through the same TREK‐1 pathway, sevoflurane enhances even neuroprotection (Cai et al., 2017; Pan et al., 2017).

Total postoperative cognitive score was almost identical to the baseline results with a difference of only a few decimals in total study population. Yet, thyroid surgery itself is a low‐risk, short to intermediate duration procedure that triggers relatively mild disturbances. We found no association between postoperative cognitive scores and the duration of anesthesia, except for logic tasks. Though POCD is more frequent after major orthopedic or cardiovascular operations (Chen et al., 2015; Wang et al., 2014) and correlates with the duration of anesthesia (Alalawi & Yasmeen, 2018), recent data show that cognitive dysfunction develops even after short surgeries and interventional procedures, such as percutaneous coronary interventions or C‐section (Bahr et al., 2022; Evered et al., 2011; Evered & Silbert, 2018).

We found a negative correlation, moderate though, between the lowest intraoperative temperature and the total postoperative and memory scores in the entire population under analysis. Interestingly, the negative impact was observed in the patients who received sevoflurane, but not the desflurane. Animal studies identified the effect fast rewarming and subsequent inflammatory response have on the neuronal injury (Wang et al., 2014). Hypothermia in humans has also been identified as increasing the risk for POCD (Gong et al., 2018). Notably, our study revealed only one hypothermic patient whose lowest recorded temperature was 34.8°C, and this patient was not the one diagnosed with the POCD. Besides, we did not record the highest temperatures, nor did we investigate the dynamic of intraoperative temperature. Thus, our data is insufficient to draw further conclusions.

According to the study protocol, the entropy levels were measured to ensure the equal depth of sleep in all the participants and to avoid anesthetic overdose, which has been identified as another risk factor of POCD (Laopaiboon et al., 2018). Likewise, cerebral hypoperfusion or hypoxia caused by hypotension or hemorrhage during surgery also promotes the development of POCD (Li et al., 2010). In our case, no episodes of hypoxia or hypotension were observed in patients under anesthesia.

### 4.1 Weaknesses and limitations of our study

One of the weaknesses of our study is that we only investigated short‐term postoperative results. Nevertheless, POCD tends to diminish over time (Wang et al., 2014). Therefore, taking into account the low incidence of POCD observed after 24 h, it is not likely that a higher incidence occurs in due time. Also, an increased incidence of ambulatory and day surgery rises the need for data about cognitive functioning in the immediate and early postoperative period since an ability to fully function physically and mentally after the surgery is closely related to patient satisfaction (Shen et al., 2022).

We did not include patients with diagnosed neurological, psychiatric or cognitive conditions, including dementia, in our study. However, we relied on medical records revealing such diagnosis. Since we did not perform any specific tests to screen for preexisting cognitive impairment, it is possible that some of the study participants had previously undiagnosed cognitive disorders.

Even though initially an equal number of participants was selected in both study arms, the final outcomes included a different number of patients (48 from the sevoflurane and 34 from the desflurane arm) as a consequence of a higher dropout ratio in the desflurane group. Higher than initially expected dropout ratio also has an impact on the statistical power of the study, since the precalculated sample size was not reached in the final analysis due to unexpected circumstances. The start of COVID‐19 pandemic and subsequent cessation of elective surgery limited our ability to enroll additional patients. Similarly, existent data on the incidence of POCD and cognitive scoring results cover high‐risk procedures, while in our case rather low differences between cognitive scoring results could be expected. Yet, performing profound cognitive testing is complicated, time and human resource‐consuming process. Therefore, it is virtually unfeasible to test extensively large numbers of patients that would be required to detect small and rare changes in cognition.

Growing evidence of genetic predisposition for cognitive impairment should be considered in similar trials. Most of our study participants were female. A menopausal loss of female sex hormones may have an impact on the cognitive function deterioration in APOE ε4 genotype female carriers, especially when inhalational anesthesia is used and memory tasks are performed (Cai et al., 2012; Müller‐Gerards et al., 2019).

## 5 CONCLUSIONS

In this study we failed to demonstrate a significant difference in postoperative cognitive changes between the patients who received either sevoflurane or desflurane during minor surgery for thyroid disease. The incidence of POCD was 1.22%. Neither age nor duration of anesthesia correlates with the total postoperative cognitive score. However, there might be an association between the total postoperative cognitive performance and the intraoperative temperature, suggesting a potential focus for future studies.

## AUTHOR CONTRIBUTIONS

VK: conceptualization, methodology, data curation, analysis, writing—original draft, and editing. EKO: analysis, supervision, writing—review, and editing. EKa: investigation, writing—original draft, and editing. IS: investigation, writing—original draft. GB: investigation, writing—original draft. GBN: investigation, writing—original draft. DJ: writing—review and editing. The final version of the manuscript has been reviewed and approved by all authors.

## FUNDING

This research received no external funding.

## CONFLICT OF INTEREST STATEMENT

The authors do not have any conflict of interest to declare regarding this manuscript.

### Peer Review

The peer review history for this article is available at https://publons.com/publon/10.1002/brb3.3017.

## ETHICS STATEMENT

The approval for the trial was obtained from the Vilnius Regional Biomedical Research Ethics Committee, Vilnius, Lithuania (Approval No. 158200‐17‐949‐453; September 12th, 2017). The principles of Declaration of Helsinki were followed. All eligible patients were provided with the information about the study and signed an informed consent form on the day of inclusion.

## CLINICAL TRIAL REGISTRATION

Trial has been registered at the institutional registry (2018 GR‐989).

## Data Availability

Collected data are not publicly available due to the sensitivity of patient health information and data protection regulations upon ethics approval. Study participants were assured that raw data would be confidential and would not be shared. Detailed trial protocol and methods are not public, but available upon a sensible request from the corresponding author.
